# Generating Visual Flickers for Eliciting Robust Steady-State Visual Evoked Potentials at Flexible Frequencies Using Monitor Refresh Rate

**DOI:** 10.1371/journal.pone.0099235

**Published:** 2014-06-11

**Authors:** Masaki Nakanishi, Yijun Wang, Yu-Te Wang, Yasue Mitsukura, Tzyy-Ping Jung

**Affiliations:** 1 Graduate School of Science and Technology, Keio University, Yokohama, Kanagawa, Japan; 2 Swartz Center for Computational Neuroscience, Institute for Neural Computation, University of California San Diego, La Jolla, California, United States of America; 3 Center for Advanced Neurological Engineering, Institute of Engineering in Medicine, University of California San Diego, La Jolla, California, United States of America; Interdisciplinary Center (IDC) Herzliya, Israel

## Abstract

In the study of steady-state visual evoked potentials (SSVEPs), it remains a challenge to present visual flickers at flexible frequencies using monitor refresh rate. For example, in an SSVEP-based brain-computer interface (BCI), it is difficult to present a large number of visual flickers simultaneously on a monitor. This study aims to explore whether or how a newly proposed frequency approximation approach changes signal characteristics of SSVEPs. At 10 Hz and 12 Hz, the SSVEPs elicited using two refresh rates (75 Hz and 120 Hz) were measured separately to represent the approximation and constant-period approaches. This study compared amplitude, signal-to-noise ratio (SNR), phase, latency, scalp distribution, and frequency detection accuracy of SSVEPs elicited using the two approaches. To further prove the efficacy of the approximation approach, this study implemented an eight-target BCI using frequencies from 8–15 Hz. The SSVEPs elicited by the two approaches were found comparable with regard to all parameters except amplitude and SNR of SSVEPs at 12 Hz. The BCI obtained an averaged information transfer rate (ITR) of 95.0 bits/min across 10 subjects with a maximum ITR of 120 bits/min on two subjects, the highest ITR reported in the SSVEP-based BCIs. This study clearly showed that the frequency approximation approach can elicit robust SSVEPs at flexible frequencies using monitor refresh rate and thereby can largely facilitate various SSVEP-related studies in neural engineering and visual neuroscience.

## Introduction

Steady-state visual evoked potential (SSVEP) is the brain’s electrical response to repetitive visual stimulation, which can be recorded from the scalp over the visual cortex, with maximum amplitude at the occipital region. In the human visual cortex, the firing of neurons synchronizes to the frequency of the stimulation and results in SSVEP, also known as a photic driving response characterized by sinusoidal-like waveforms at the stimulus frequency and its harmonics [1]. The frequency components in the SSVEP signals remain constant in amplitude over time, and therefore the stimulus frequency can be reliably recognized based on the measurement of SSVEP in the frequency domain. Due to the robust frequency character of the SSVEP, the frequency tagging technique, which encodes multiple visual targets with different flickering frequencies, has been widely used in the fields of visual neuroscience and neural engineering [2], [3]. For example, a large number of visual attention studies used frequency-tagged SSVEPs to investigate the attentional modulation in the visual cortex [4]–[7]. Recently, the frequency tagging technique has also been introduced to Electroencephalogram (EEG)-based brain-computer interfaces (BCIs), which can translate intentional brain activities to commands to control an external device [8].

The SSVEP-based BCI has attracted much attention for its advantages of little user training, ease of use, and high information transfer rate (ITR) [9]–[14]. Among various coding methods [2], [14], frequency coding is the most convenient way to implement an SSVEP-based BCI. In such a system, users are asked to fixate on one of multiple visual stimuli flickering at different frequencies, and the target stimulus can be identified through identifying the dominant frequency of the SSVEPs. The cortical magnification theory [15] is the basic principle of an SSVEP-based BCI. In the visual cortex, large areas are allocated to process the central visual field, so the visual acuity is highest when the stimulus is located in the center of the visual field. Therefore, the amplitude of SSVEP increases enormously as the stimulus is moved closer to the central visual field. In an SSVEP-based BCI, different commands, which are represented by frequency-tagged SSVEPs, can be produced by directly looking at one of multiple frequency-coded stimuli.

The visual stimulator plays an important role in the success of an SSVEP-based BCI [2]. Visual stimuli can be presented using flashing light-emitting diodes (LEDs) or flickers on a computer monitor. The stimulation parameters such as the amount, color, pattern, size, and position of visual stimuli can be configured flexibly on a computer monitor. However, the number of frequencies that can be presented is always limited by the refresh rate of a monitor. In the conventional constant-period approach, the number of frames in a flickering period is a constant for each stimulating frequency. For instance, a monitor with a 60 Hz refresh rate can only present flickers at 7.5 Hz (8 frames per period), 8.57 Hz (7 frames per period), 10 Hz (6 frames per period), 12 Hz (5 frames per period), and 15 Hz (4 frames per period) around the EEG alpha band (8–13 Hz), where the SSVEP signal has the highest amplitude [1]. In this way, complicated applications such as a phone-dialing program [13], which requires at least 12 targets (10 digits, Backspace, and Enter), cannot be implemented. Furthermore, the increase of the number of commands in an SSVEP-based BCI can generally lead to an increase of ITR [2]. Therefore, it is of great importance to find a solution to realize the presentation of visual flickers with a high frequency resolution on a computer monitor. The limitation of target numbers on a computer monitor has hindered practical applications of current SSVEP-based BCI systems. Noticeably, with rapid advances in the mobile technology such as mobile phone/tablet, a general framework for presenting SSVEP stimuli on the screen of the mobile devices is in need. A mobile visual stimulator can significantly facilitate the implementation of mobile BCI systems [16]–[19].

Recently, Wang et al. (2010) proposed an approximation method to realize visual flickers with a high frequency resolution using a computer monitor [20]. Any frequency (lower than half of the refresh rate) can be approximated by using variable frequencies in different stimulating periods. Using this approach, a 16-target SSVEP-based BCI system (frequency range: 9–12.75 Hz, frequency resolution: 0.25 Hz) was implemented and obtained an average ITR of 75.4 bits/min. Although the approximation approach was further proved by several studies [21]–[24], it has not been widely used in the recent SSVEP-related studies in BCI and visual neuroscience. The main reason is that a direct comparison between the SSVEPs elicited by the conventional constant-period approach and the approximation approach is missing. Therefore, it remains unclear whether or how the approximation approach would change the signal characteristics of the SSVEPs. The lack of a quantitative comparison between SSVEPs elicited by the two approaches poses serious doubts about the reliability of the approximation approach in many research topics where signal characters such as amplitude, signal-to-noise ratio (SNR), phase and latency, and scalp distribution need to be very accurate. These signal parameters can accurately characterize the SSVEPs and thereby play important roles in assessing SSVEPs in BCI and visual neuroscience studies. For example, the phase coding method has been widely used in the SSVEP-based BCI systems [2]. If the phase of the SSVEPs elicited by the approximation approach can be proved stable, the frequency and phase mixed coding method proposed by Jia et al. (2011) can be further improved using the approximation approach [25]. Besides, in visual neuroscience research, there are many situations that require accurate measurement of signal characters of SSVEPs at multiple frequencies [3], [4]. In these circumstances, the feasibility of the approximation approach highly depends on the stability and robustness of the elicited SSVEPs. To answer these questions, this study proposes to compare the amplitude, SNR, phase and latency, scalp distribution, and frequency detection accuracy of SSVEPs elicited using the two approaches with a CRT monitor at two refresh rates (75 Hz and 120 Hz). In this way, SSVEPs at 10 Hz and 12 Hz under the two refresh rates can be measured separately to represent the approximation approach (under 75 Hz) and the constant-period approach (under 120 Hz) respectively. A flat-panel monitor typically has very limited options in refresh rates (e.g., 50 Hz or 60 Hz), which cannot present multiple stimulus frequencies using both approaches. Therefore, the employment of a monitor with more adjustable refresh rates is crucial for implementing a quantitative comparison of the two approaches. Specifically, this study aims to perform a quantitative comparison to validate the feasibility of the approximation approach in eliciting robust SSVEP signals. In addition, to further prove the efficacy of the approximation method in implementing a high-speed SSVEP-based BCI, this study demonstrated an eight-target BCI system using stimulus frequencies in a relatively wide range of 8–15 Hz using an LCD monitor.

## Materials and Methods

### 1 Ethics Statement

The offline experiment was approved by the Human Research Protections Program of the University of California San Diego. The simulated online BCI experiment was approved by the Research Ethics Committee of Keio University. All participants were asked to read and sign an informed consent form before participating in the study.

### 2 Visual Stimulus Design

**Figure 1:**
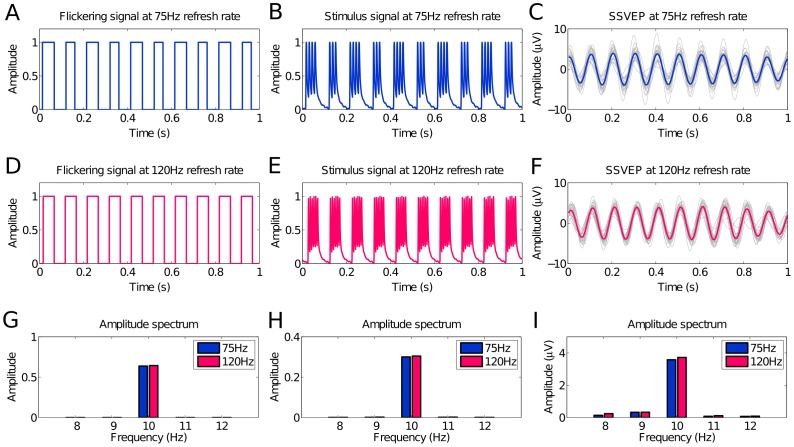
Time series and amplitude spectra of stimulus signal and SSVEPs. Time series sequences of (A) flickering signal, (B) real stimulus signal and (C) elicited SSVEPs by 10 Hz stimuli presented on a CRT monitor with a 75 Hz refresh rate, and (D) flickering signal, (E) real stimulus signal and (F) elicited SSVEPs by 10 Hz stimuli under a 120 Hz refresh rate. Amplitude spectra of (G) flickering signal, (H) real stimulus signal and (I) elicited SSVEPs by 10 Hz stimuli under the 75 Hz and 120 Hz refresh rates.

In the conventional SSVEP-based BCIs using a computer monitor, a stimulating period of a visual flicker consists of a constant number of frames. For instance, the 10 Hz visual flicker under a 60 Hz refresh rate can be produced by reversing the stimulus pattern between white and black every three frames as ‘111000111000111000…’, where ‘1’s and ‘0’s represent white and black respectively. In this way, it is impossible to realize the frequencies by which the refresh rate is not dividable (e.g., an 11 Hz flicker under a 60 Hz refresh rate) because the white/black reversal should occur every 2.73 frames. The approach proposed by Wang et al. (2010) can realize such flickering frequencies by approximating a frequency with variable number of frames in a stimulating period [20]. For instance, an 11 Hz flicker can be realized by interleaving five and six frames in a period as ‘1110001110011100011100111…’. In other words, an 11 Hz flicker can be approximated by mixing stimulating periods of 10 Hz and 12 Hz, which can be realized using the constant-period approach. More generally, the stimulus sequence 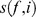 at frequency *f* can be described as follows:
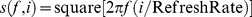
(1)where square() generates a 50% duty cycle square wave with levels 0 and 1, and *i* indicates the frame index. In this way, a flicker at any frequency up to half of the monitor refresh rate can be realized. Fig. 1A and Fig. 1D show the theoretical flickering signals at 10 Hz under the 75 Hz and 120 Hz refresh rates generated by (1). The 10 Hz stimulus signal under the 75 Hz refresh rate comprises interleaved seven- and eight-frame long periods. With the 120 Hz refresh rate, the stimulus signal has a constant period of 12 frames.

### 3 Offline Experiment

#### 3.1 Data acquisition

The offline experiment was designed to compare the signal characteristics of SSVEPs elicited by the constant-period approach and the approximation approach. In this experiment, the visual stimulus (a 5×5 cm flicker) was rendered at the center of a ViewSonic P810 21-inch CRT monitor (ViewSonic Corp.) with a refresh rate of 75 Hz and 120 Hz respectively. The stimulus frequencies ranged from 9 Hz to 13 Hz with a 1 Hz interval. The frequencies of the stimuli were in the alpha band because BCI using SSVEPs in this frequency range can obtain higher classification performance than other frequency bands [11]. Here, the visual stimuli at 10 Hz and 12 Hz under the 120 Hz refresh rate were produced by the constant-period approach. Other frequencies under the 120 Hz refresh rate and all frequencies under the 75 Hz refresh rate were generated by the approximation approach. Therefore, the 10 Hz and 12 Hz SSVEPs under the two refresh rates are appropriate for comparing the two approaches. In addition, data of all five frequencies under each refresh rate were put together for exploring the difference between the two refresh rates. The stimulation program was developed in Microsoft Visual C++ using the Microsoft DirectX 9.0 framework.

The EEG data were measured from ten healthy male adults with normal or corrected-to-normal vision. All subjects were asked to read and sign an informed consent form approved by the UCSD Human Research Protections Program before participating in this experiment. The subjects were seated in a comfortable chair 35 cm away from the monitor in a dark room. A chin rest was used to help them maintain head position. Each subject was instructed to gaze at ten visual stimuli (five frequencies×two refresh rates) for 30 seconds each in a single run sequentially, and perform a total of four runs. Subjects were instructed to avoid eye blinks during the 30-second gaze duration. To avoid visual fatigue, there was a several-second rest after each stimulus and a several-minute rest after each run. In each run, the ten stimuli were presented in a random order. EEG data were recorded using Ag/AgCl electrodes from 256 locations distributed over the entire head using a BioSemi ActiveTwo EEG system (Biosemi, Inc.). Electrode locations were measured with a 3-D digitizer system (Polhemus, Inc.). EEG signals were amplified and digitized at a sampling rate of 2048 Hz. All electrodes were with reference to the CMS electrode close to Cz. Event triggers (i.e., an event code every 4 seconds) generated by the stimulus program were sent from the parallel port of the computer and recorded on an event channel synchronized to the EEG data.

In addition to the EEG data, 60s-long flickering signals for all stimuli were recorded separately using a phototransistor (PNZ108CLR, Panasonic Corp.) attached to the surface of the monitor and a customized biosignal recording system. The stimulus signals were digitized with a sampling rate of 1000 Hz.

#### 3.2 Data analysis

The 256-channel EEG data were first down-sampled to 256 Hz, and then band-pass filtered between 5–30 Hz to remove SSVEP unrelated frequency components. Six 4s-long EEG epochs were extracted from each 30s-long trial along event triggers generated by the stimulus program. For each stimulus frequency (from 9 Hz to 13 Hz) under each refresh rate, the epochs from all four runs (six epochs in each run) were put together to form a dataset of 24 epochs.

To explore the signal characteristics of the SSVEPs elicited by the two approaches, this study first compared the amplitude, SNR, and latency of single-channel SSVEPs. To be noticed, the 10 Hz and 12 Hz SSVEPs under the two refresh rates were used to represent the two approaches. The amplitude spectrum 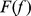 was calculated by taking the absolute value of the fast Fourier transform (FFT):
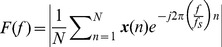
(2)where ***x***(*n*) is single-channel EEG signals, *f* is the stimulation frequency, *f_s_* is the sampling rate, and *N* is the data length. In this study, *N* is set to 1024 (i.e., 4 seconds). The SNR of SSVEPs was defined as the ratio of the amplitude of the SSVEP at the stimulating frequency to the mean amplitude of the background EEG activities within the neighboring frequency bands:
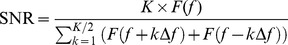
(3)where 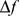 is the frequency resolution in the amplitude spectrum. In this study, 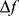 is 0.25 Hz and *K* is set to 12. The phase of SSVEPs 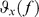 can be calculated as follows:



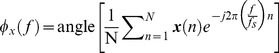
(4)Then, the phase difference (in π radians) between SSVEPs at two stimulating frequencies (*f*_1_ and *f*_2_) is defined as 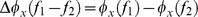. The response latency *t* (in milliseconds) between the stimulus and the SSVEP can be derived by measuring phase as a function of stimulating frequency and estimating the slope of the curve [26], [27]:
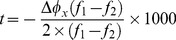
(5)


In addition, this study also observed the scalp distribution of the amplitude of SSVEPs. The scalp topography maps based on multichannel SSVEP amplitudes were illustrated using the TOPOPLOT function in EEGLAB toolbox [28]. The difference map between the two refresh rates were used to detail the spatial distribution of the amplitude difference.

To compare the frequency detection accuracy between the two approaches, this study performed offline classification of two frequencies (10 Hz and 12 Hz) and all five frequencies (9–13 Hz) using FFT- and canonical correlation analysis (CCA)-based methods [29]. Note that, the visual stimuli at any frequency under the 75 Hz refresh rate and the 9 Hz, 11 Hz, and 13 Hz stimuli under the 120 Hz refresh rate were rendered using the approximation approach. Therefore, the performance of the two approaches can be simply evaluated by comparing the accuracy of the two-frequency classification. Although the comparison of the five-frequency classification under the two refresh rates mixed the two approaches, it is helpful for optimizing an SSVEP-based BCI with regard to the refresh rate. In the FFT-based method, the target stimulus can be identified through detecting the frequency peak in the amplitude spectrum. Although the FFT-based method has been widely used in SSVEP-based BCIs, recent studies reported that the CCA method can significantly improve the SNR of the SSVEP signals [29], [30]. In SSVEP detection, the CCA method is as efficient as other multi-channel methods such as the minimum energy combination (MEC) method [21], [31]. CCA is a statistical way to measure the linear relationship between two multidimensional variables, which may have some underlying correlation. Considering two multidimensional variable ***X***, ***Y*** and their linear combinations ***x*** *=* ***X*** *^T^W_x_* and ***y*** *=* ***Y*** *^T^W_y_*, CCA finds the weight vectors, *W_x_* and *W_y_*, which maximize the correlation between ***x*** and ***y*** by solving the following problem:
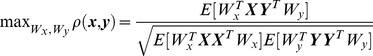
(6)


The maximum of *ρ* with respect to *W_x_* and *W_y_* is the maximum canonical correlation. Projections onto *W_x_* and *W_y_* are called canonical variants. Here, ***X*** refers to the set of 4s-long multi-channel EEG signals and ***Y*** refers to the set of reference signals that have the same length as ***X***. To avoid overfitting, sixteen electrodes over the occipital region were selected for CCA. The reference signals ***Y*** *_f_* are set as
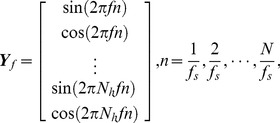
(7)where *f* is the target frequency, *N_h_* is the number of harmonics, and *N* is the number of sampling points. To recognize the frequency of the SSVEPs, CCA calculates the canonical correlation between the multi-channel EEG signals and the reference signals at each stimulus frequency. The frequency of the reference signals with the maximal correlation was selected as the frequency of the SSVEPs.

### 4 Simulated Online BCI Experiment

Our previous study using the approximation approach was tested with only three subjects [20]. To further validate the efficacy and generalization of the approximation approach across different people, this study conducted a simulated online BCI experiment [25] with more subjects. In the experiment, a Dell S2409W 24-inch LCD monitor (Dell Inc.) with a 75 Hz refresh rate was used to present eight stimuli (each with a size of 3×3 cm) with flickering frequencies from 8 to 15 Hz with a 1 Hz interval. The paradigm can be used to implement an eight-target cursor control system. The stimulus program was developed under MATLAB (Mathworks Inc.) using the Psychophysics Toolbox extensions [32].

Ten healthy adults (8 males and 2 females, mean age: 23 years) with normal or corrected-to-normal vision participated in the experiment. All subjects signed an informed consent form approved by the Research Ethics Committee of Keio University before participating in the experiment. The EEG signals were recorded by four electrodes located at the occipital area (Pz, O1, Oz, and O2) using the g. USBamp (g.tec medical engineering GmbH) with a sampling rate of 256 Hz. The subjects were seated in a comfortable chair 70 cm away from the monitor in a dark room. They were asked to input a sequence with eight commands in a task, and to repeat the task 15 times in the experiment. At the beginning of each command, a red rectangle marker (3×3 cm) appeared at the position of the target stimulus. Subjects were asked to shift their gaze to the target within a duration of 0.5 second. After that, all stimuli started to flicker simultaneously for one second on the monitor. The 1s-long EEG data synchronized to the visual stimuli were used for target identification. The order of targets was randomized in the task sequence.

The recorded EEG epochs were classified by the CCA-based method. The simulated online BCI performance is evaluated by ITR calculated as follows [8]:
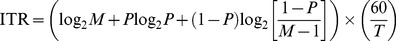
(8)where *M* is the number of targets, *P* is the accuracy of frequency detection, and *T* (seconds/selection) is the average time for a selection. In this study, *M* is 8 and *T* is 1.5 second (1s for target gazing and 0.5s for gaze shifting). At a speed of 40 selections per minute, the proposed system could obtain a maximum ITR of 120 bits/min.

## Results

### 1 Temporal Waveforms and Amplitude Spectra of Stimulus Signal and SSVEPs

Fig. 1A and Fig. 1D show the theoretical stimulus signals at 10 Hz under the 75 Hz and 120 Hz refresh rates generated by the approximation approach and the constant-period approach respectively. Fig. 1B and Fig. 1E show the real stimulus signals recorded by the phototransistor. The scanning signal of the monitor is clearly shown in each white frame. Fig. 1C and Fig. 1F show the time series of averaged SSVEPs elicited by the two stimulus signals (Fig. 1B and Fig. 1E) from a representative subject. The amplitude spectra of the two theoretical stimulus signals show very comparable peak amplitudes at the stimulation frequency. The amplitude spectra of the recorded stimulus signal and SSVEPs (Fig. 1H and Fig. 1I) both show comparable peak amplitudes at the stimulation frequency under the two refresh rates. These figures clearly demonstrate that the frequency of the SSVEPs elicited by the two approaches match the stimulus signal well.

**Figure 2:**
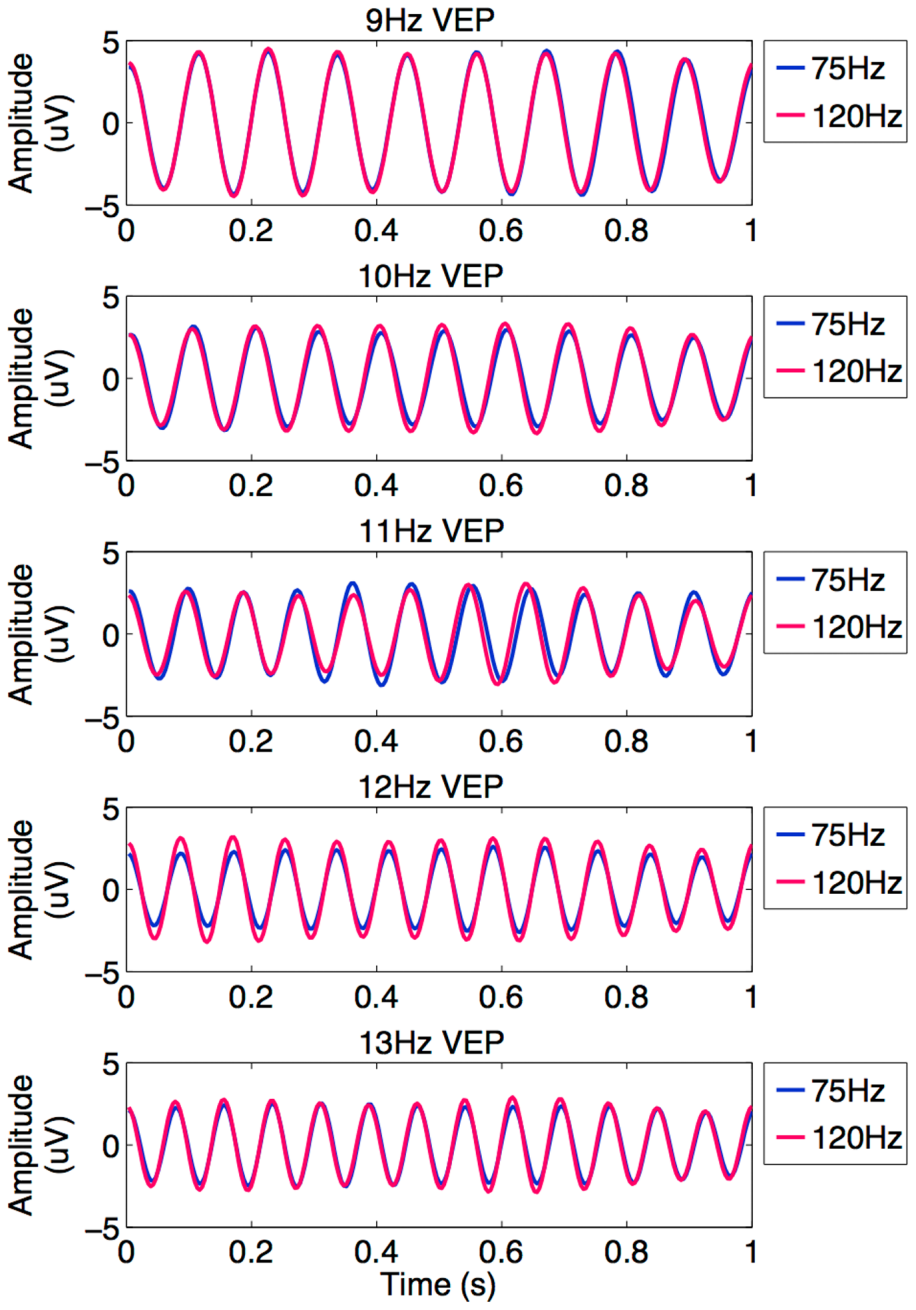
Grand average SSVEP waveforms elicited at 9–13 Hz stimuli under 75 Hz and 120 Hz refresh rates.

Fig. 2 shows the averaged temporal waveforms of elicited SSVEPs across all subjects for all five frequencies under the 75 Hz and 120 Hz refresh rates. The signals were recorded from the Oz electrode. To better observe the amplitudes and phases in SSVEPs, the SSVEP signals were band-pass filtered between [*f*–2 *f*+2], where *f* is the stimulating frequency, to remove the background EEG activities. For all conditions, the grand average SSVEP signal is a near-sinusoidal waveform with the same frequency as the stimulus signal. For each frequency, the frequency components in the SSVEP signals have stable amplitude and phase over time, which are very comparable under the 75 Hz and 120 Hz refresh rates. A more detailed comparison using statistical analysis will be described in the next two subsections.

### 2 Amplitude and SNR

**Figure 3:**
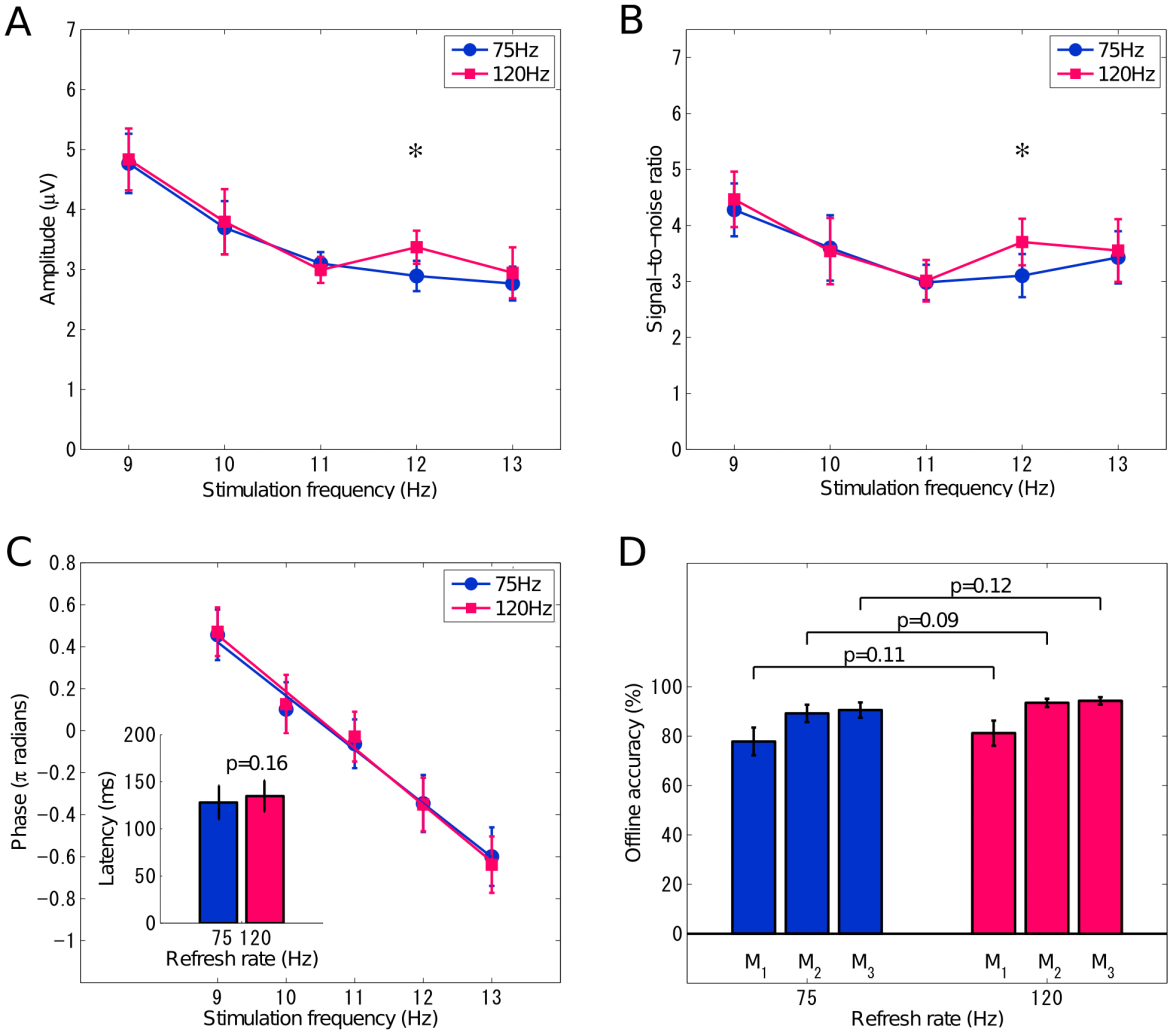
Signal characteristics and offline classification accuracy comparison between 75 Hz and 120 Hz refresh rates. Averaged (A) amplitudes (B) SNRs of elicited SSVEPs at each stimulus frequency across all subjects, (C) averaged phases in *π* radians, and latencies across subjects, and (D) averaged classification accuracy across subjects under 75 Hz and 120 Hz refresh rates using FFT-based method (M_1_) and CCA-based methods (M_2_: with the fundamental harmonic in the reference signals; M_3_: with the fundamental and second harmonics in the reference signals). Error bars indicate standard errors. The asterisk indicates a significant difference between 75 Hz and 120 Hz (p<0.001).

Fig. 3A shows the amplitude of elicited SSVEPs at the Oz electrode for all stimulus frequencies under the two refresh rates. From 9 Hz to 13 Hz, the amplitude of SSVEPs decreased following the increase of the stimulus frequency. The averaged amplitudes of SSVEPs at 10 Hz under the 75 Hz and 120 Hz refresh rates, realized by the approximation approach and the constant-period approach, were 3.70 *µV* and 3.80 *µV*, and those at 12 Hz were 2.89 *µV* and 3.37 *µV*. A paired t-test shows a significant difference between the amplitudes of SSVEPs at 12 Hz elicited by the two stimulation approaches (p<10^−4^). However, there is no significant difference between the two approaches at 10 Hz (p = 0.70). The amplitudes of SSVEPs at the other frequencies using the approximation approach were comparable under the two refresh rates (75 Hz vs. 120 Hz, 9 Hz: 4.79 *µV* vs. 4.83 *µV*, 11 Hz: 3.10 *µV* vs. 2.99 *µV*, 13 Hz: 2.77 *µV* vs. 2.95 *µV*), and there was no significant difference between the two refresh rates (9 Hz: p = 0.77, 11 Hz: p = 0.19, 13 Hz: p = 0.34).

Fig. 3B shows the SNR of SSVEPs for all stimulus conditions. The difference of SNR between the two refresh rates is consistent to that of the SSVEP amplitude. The averaged SNR across subjects at 12 Hz under the 120 Hz refresh rate was significantly higher than that under the 75 Hz refresh rate (75 Hz: 3.11, 120 Hz: 3.71, p<0.001). There was no significant difference between the two approaches at 10 Hz (75 Hz: 3.60, 120 Hz: 3.54, p = 0.82). For the other stimulus frequencies, the SNRs of SSVEPs elicited by the approximation approach under the two refresh rates are very similar (75 Hz vs. 120 Hz, 9 Hz: 4.28 vs. 4.47, p = 0.35, 11 Hz: 2.98 vs. 3.01, p = 0.73, 13 Hz: 3.43 vs. 3.55, p = 0.49).

### 3 Phase and Latency

Phase and latency were measured using the SSVEPs recorded from the Oz electrode. The averaged phases across subjects were plotted in Fig. 3C as a function of stimulus frequency for each refresh rate. Under each refresh rate, the frequency-phase curve fits a linear model, indicating that the latency of SSVEP is a constant. Fig. 3C also shows the latency estimated by the slope of the linear regression line. The latency of the SSVEPs elicited under the 75 Hz and 120 Hz refresh rates was 128 ms and 135 ms respectively. The estimated latencies are consistent with results in previous studies using the constant-period approaches [33], [25]. A paired t-test indicates that there is no significant difference between the latencies of SSVEPs elicited under the two refresh rates (p = 0.16).

### 4 Scalp Distribution

**Figure 4:**
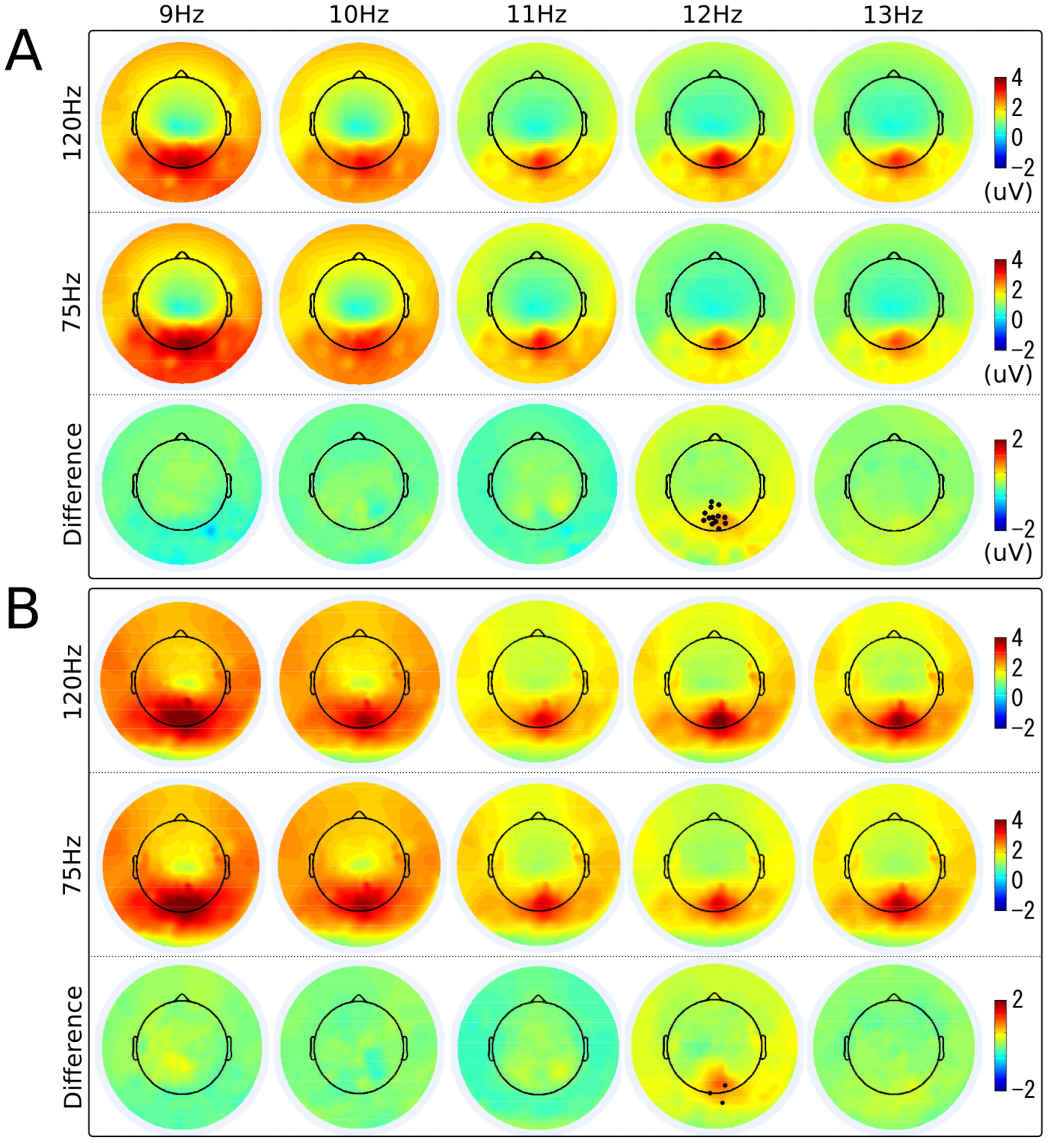
Scalp topographies of amplitude and SNR of SSVEPs under 75 Hz and 120 Hz refresh rates. Scalp topographies of (A) the amplitudes and (B) the SNRs of SSVEPs at each stimulation frequency under the 120 Hz (top) and 75 Hz (middle) refresh rate, and their difference (bottom). The electrodes with significantly different amplitudes elicited by two different refresh rates are marked with black dots (p<10^−4^).

Fig. 4 illustrates the scalp topographies of the amplitude and the SNR of SSVEPs at all stimulus frequencies under 120 Hz and 75 Hz and their difference. For all stimulus conditions, the scalp distributions were very comparable, showing maximum amplitudes at the electrodes over the occipital area. From 9 Hz to 13 Hz, the fading of map colors indicates that the amplitude of SSVEPs decreased following the increase of the stimulus frequency. As described in Section 3.2, the amplitude of SSVEPs at 12 Hz has a significant difference between the two refresh rates. The difference of scalp maps based on the amplitude and SNR at 12 Hz consistently shows a group of 13 and 3 electrodes at the occipital region with significant difference between the two refresh rates (p<10^−4^). Furthermore, the difference map at 12 Hz has a similar scalp distribution as the original amplitude maps, indicating that the amplitude difference comes from the amplitude change of brain sources in the visual cortex. There was no significant difference in the amplitude and SNR topographies under other conditions.

### 5 Offline Frequency Detection Accuracy

Table 1 lists the offline detection accuracy for the two-frequency (10 Hz and 12 Hz) classification using the FFT- and CCA-based methods. Three methods including the FFT-based method (M_1_), the CCA-based method with the fundamental harmonic (M_2_), and the CCA-based method with the fundamental and second harmonics (M_3_) were used to estimate the classification accuracy. Classification accuracy of the constant-period approach is higher than the approximation approach, which is consistent with the results of the amplitude and SNR comparisons at 12 Hz. The FFT-based method obtained averaged accuracy of 91.72% and 92.30% under the 75 Hz and 120 Hz refresh rates without a significant difference (p = 0.58). The CCA-based method improved the averaged detection accuracy (75 Hz vs. 120 Hz, M_2_∶97.33% vs. 98.96%, M_3_∶97.95% vs. 99.37%). The difference between the two approaches is also not statistically significant (M_2_: p = 0.22; M_3_: p = 0.29). The involvement of the second harmonic in CCA improved the classification accuracy. However, since all subjects reached very high accuracy using the CCA-based methods, the difference was not statistically significant (75 Hz: p = 0.08; 120 Hz: p = 0.17). Table 2 shows the confusion matrix. The FFT-based method showed higher accuracy at 12 Hz, whereas the CCA-based method presented higher accuracy at 10 Hz. These results showed the independence between the FFT- and CCA- based methods.

**Table 1:** Frequency detection accuracy (%) in two-class classification (10 Hz vs. 12 Hz).

Subject	M_1_		M_2_		M_3_	
	75 Hz	120 Hz	75 Hz	120 Hz	75 Hz	120 Hz
s1	79.17	86.96	100.00	100.00	100.00	100.00
s2	68.75	67.35	93.75	95.92	95.83	99.37
s3	100.00	100.00	100.00	100.00	100.00	100.00
s4	89.80	91.67	85.71	97.92	87.76	100.00
s5	97.92	95.74	97.92	100.00	97.92	100.00
s6	100.00	97.87	100.00	97.87	100.00	97.87
s7	100.00	100.00	100.00	100.00	100.00	100.00
s8	100.00	98.00	100.00	100.00	100.00	100.00
s9	83.67	87.50	95.92	97.92	97.96	97.92
s10	97.92	97.92	100.00	100.00	100.00	100.00
Mean±std	91.72±3.50	92.30±3.16	97.33±1.46	98.96±0.46	97.95±1.22	99.37±0.31

**Table 2:** Confusion matrix in two-class classification (10 Hz vs. 12 Hz).

	M_1_				M_2_				M_3_			
	75 Hz		120 Hz		75 Hz		120 Hz		75 Hz		120 Hz	
	10 Hz	12 Hz	10 Hz	12 Hz	10 Hz	12 Hz	10 Hz	12 Hz	10 Hz	12 Hz	10 Hz	120 Hz
10 Hz	90.10	9.90	90.71	9.29	100	0.00	100	0.00	100	0.00	100	0.00
12 Hz	6.67	93.33	6.28	93.72	5.38	94.62	2.07	97.93	4.15	95.85	1.23	98.77

Fig. 3D shows the averaged accuracy for the five-frequency classification across all subjects. As shown in the figure, the accuracies obtained by M_1_ were 77.84% and 81.19% (p = 0.11) under the 75 Hz and 120 Hz refresh rates, respectively. The CCA-based methods improved the classification accuracy (75 Hz vs. 120 Hz, M_2_∶89.20% vs. 93.46%, M_3_∶90.55% vs. 94.27%). The second harmonic in CCA improved the classification accuracy under both refresh rates. The difference was significant under the 75 Hz refresh rate (p = 0.03), whereas the difference was not significant under the 120 Hz refresh rate (p = 0.13). The difference between the two refresh rates was not significant (M_2_: p = 0.09; M_3_: p = 0.12). These results indicate that classification performance under the 120 Hz refresh rate is slightly higher than the 75 Hz refresh rate. However, the difference is not statistically significant.

### 6 Online BCI Performances

Table 3 summarizes BCI performance for all subjects in the simulated online experiment. An averaged ITR of 95.0±20.9 bits/min was obtained in the simulated online experiment. The classification accuracy is 91.0±9.0% across all subjects. Two subjects (s6 and s9) had classification accuracy of 100%, which led to an ITR of 120 bits/min. To our knowledge, an average ITR of 95.0 bits/min is the highest ITR reported in the SSVEP-based BCI systems [20], [29]. Table 4 shows the confusion matrix. The peak accuracy appeared around 10–12 Hz, which is consistent to previous SSVEP-based BCI studies [11]. These results further prove the feasibility of the approximation approach in eliciting robust SSVEP signals within a wide range of stimulating frequencies (8–15 Hz) in an online BCI paradigm.

**Table 3:** BCI performance in the simulated online experiment.

Subject	Accuracy (%)	ITR (bits/min)
s1	90.83	92.03
s2	96.67	107.82
s3	84.17	77.01
s4	92.50	96.21
s5	95.00	102.93
s6	100.00	120.00
s7	94.17	100.62
s8	86.67	82.37
s9	100.00	120.00
s10	70.00	51.06
Mean±std	91.00±9.00	95.00±20.90

**Table 4:** Confusion matrix in the simulated online experiment.

Input	Output of the simulated online test							
	8 Hz	9 Hz	10 Hz	11 Hz	12 Hz	13 Hz	14 Hz	15 Hz
8 Hz	89.33	2.00	0.67	0.67	4.00	2.00	0.67	0.67
9 Hz	1.33	91.33	2.67	0.67	0.00	2.67	0.67	0.67
10 Hz	1.33	0.00	97.33	0.00	0.67	0.00	0.67	0.00
11 Hz	2.00	0.00	2.00	93.33	1.33	0.00	1.33	0.00
12 Hz	0.00	0.67	0.00	1.33	98.00	0.00	0.00	0.00
13 Hz	8.00	1.33	0.00	0.00	0.00	90.67	0.00	0.00
14 Hz	3.33	1.33	1.33	0.67	4.00	6.67	82.00	0.67
15 Hz	4.00	1.33	0.00	0.00	2.00	1.33	5.33	86.00

## Discussions

### 1 Signal Characteristics of SSVEPs

The stimulus presentation based on the approximation approach has been proved efficient to elicit SSVEPs with a high frequency resolution for the SSVEP-based BCI [20]. However, no study has directly compared the amplitude and the SNR of SSVEPs elicited by the constant-period and the approximation approaches. Therefore, the exact efficacy of the approximation approach in eliciting robust SSVEPs remains unknown. This study measured SSVEPs elicited by the constant-period approach and the approximation approach using a CRT monitor with the 75 Hz and 120 Hz refresh rates. The 10 Hz and 12 Hz SSVEPs under the two refresh rates represented the two approaches respectively. This study compared amplitude, SNR, phase and latency, and scalp distribution of the SSVEPs elicited by the two approaches. All these parameters of the SSVEPs at 10 Hz are comparable between the two approaches. Interestingly, the amplitude of the SSVEPs at 12 Hz under the 120 Hz refresh rate is statistically significantly higher than that under the 75 Hz refresh rate (p<10^−4^). It might be explained by the generation of additional SSVEP signal at 12 Hz elicited by the scanning signal of the monitor at 120 Hz. A previous study found that the subharmonic components can be observed in SSVEPs at some selective frequencies [34]. In this sense, 12 Hz might be a specific subharmonic frequency for the SSVEPs elicited by the refreshing signal at 120 Hz. Further investigations are required to explore the underlying neural mechanism of this effect. The comparison of the SSVEPs at three other frequencies (9 Hz, 11 Hz, and 13 Hz) using the approximation approach indicates that there is no significant difference between the two refresh rates. As shown in Fig. 3A, since the approximation approach under the 75 Hz refresh rate generates SSVEPs following a smooth distribution along stimulation frequencies (9–13 Hz), the observed SSVEP difference at 12 Hz could be attributed to an increase of SSVEP amplitude using the constant-period approach (under the 120 Hz refresh rate). Further investigations are required to prove this hypothesis. Taken together, this study validates the feasibility of the approximation approach in eliciting robust SSVEP signals at flexible frequencies.

This study only focused on the fundamental harmonic of SSVEPs in assessing signal characteristics. Since SSVEP harmonics have been widely adopted in frequency detection [2], it is also interesting to investigate the harmonics of SSVEPs elicited by stimuli using monitor refresh rate. Interestingly, the results of the second harmonics were different from that of the fundamental frequency. The amplitudes of the second harmonics of 9 Hz and 13 Hz were comparable under the two refresh rates (i.e. no significant difference in 75 Hz vs. 120 Hz, 9 Hz: 2.65 *µV* vs. 2.63 *µV*, p = 0.87; 13 Hz: 1.11 *µV* vs. 1.12 *µV*, p = 0.82). However, the second harmonics of the other three frequencies showed significantly different amplitudes (75 Hz vs. 120 Hz, 10 Hz: 1.87 *µV* vs. 2.27 *µV*, p = 0.01; 11 Hz: 1.52 *µV* vs. 1.83 *µV*, p = 0.02; 12 Hz: 1.29 *µV* vs. 1.62 *µV*, p = 0.02). One explanation might be that the interaction between the subharmonics of 120 Hz (e.g., 20 Hz, 24 Hz) and the stimulus frequency (10–12 Hz) enhance the second harmonics of SSVEPs at these frequencies. In a recent study [35], we found that the refresh rate-based stimulation approach also elicits SSVEPs at other frequencies that are termed interference frequencies, which are derived from the interaction between the stimulation frequency and the refresh rate. Since the amplitude difference is relatively small, further investigations are required to validate this finding.

### 2 Classification Performance

This study compared the classification performance between the SSVEPs elicited by the approximation approach and the constant-period approach using a binary classification of the SSVEPs at 10 Hz and 12 Hz. The averaged classification accuracy of the approximation approach was slightly lower than that of the constant-period approach but with no significant difference (FFT: 91.72% vs. 92.30%, p = 0.58; CCA: 97.33% vs. 98.96%, p = 0.22). In addition, this study performed a five-class classification (9–13 Hz) under the two refresh rates and again showed that the averaged classification accuracy under 75 Hz was slightly lower than that under 120 Hz but the difference was not significant. These results indicate that the two stimulus presentation methods can achieve comparable BCI performance. Therefore, the approximation approach can satisfy the requirement of a large number of visual stimuli in an SSVEP-based BCI. Interestingly, the 120 Hz refresh rate seems to be able to enhance the 12 Hz SSVEPs, and thus leads to higher classification accuracy. From this perspective, the frequencies that can be realized using the constant-period approach should be first considered in an SSVEP-based BCI.

This study evaluated the online BCI performance obtained in the current simulated online test using the approximation approach. The classification accuracy of the simulated online test is very high across all subjects (91.0±9.0%). Compared with a similar eight-target BCI system that obtained a communication speed at 3.4 seconds per target in [36], the communication speed in this study is significantly higher (1.5 seconds per target) due to higher SNR of SSVEPs elicited by the approximation approach in the alpha frequency range (8–15 Hz with a 1 Hz interval). Theoretically, the ITR could be further improved by increasing the number of targets. The summary of these systems indicates that the approximation approach is very flexible with regard to rendering device, refresh rate, and the number of visual stimuli.

### 3 Stimulation Frequency and Refresh Rate

This study used 10 and 12 Hz visual stimuli, which have been widely used in previous SSVEP studies that used the constant-period approach, to compare the two presentation approaches. In practice, it is impossible to implement 9 Hz, 11 Hz, and 13 Hz using the constant-period approach under regular monitor refresh rates (e.g., 60 Hz, 75 Hz, 120 Hz). Although the fact that 12 Hz condition showed a significant difference in SNR and amplitude across refresh rates made the comparison less thoroughly, the present results already provide sufficient evidence to the robustness of the approximation approach. To improve the comparison study, a light-emitting diode (LED)-based stimulator could be developed for comparing more frequencies by simulating different monitor refresh rates (e.g., a 90 Hz refresh rate for implementing a 9 Hz flicker using the constant-period approach).

In this study, the other three frequencies, 9 Hz, 11 Hz, and 13 Hz, were generated by the approximation approach under the two refresh rates. Except for 12 Hz under the 120 Hz refresh rate, the amplitude and SNR curves follow distinct patterns across the five frequencies (9–13 Hz). In addition, the phase and latency analysis showed very consistent results across all five frequencies. To some extent, these findings also prove the robustness of the approximation approach as compared to the constant-period approach.

This study only focused the stimulus frequencies within the EEG alpha frequency band, which has been widely used in the SSVEP-based BCIs [2]. The approximation approach is also applicable to other frequency bands in EEG signals. Several recent studies reported the employment of SSVEPs with higher frequencies (>20 Hz) in BCI studies [37], [38]. The high-frequency SSVEPs can improve the comfortableness of the BCI system due to its advantage of less visual fatigue. Compared with the alpha frequency band, the implementation of high-frequency flickering stimuli is more seriously limited by the refresh rate. For example, the constant-period approach is only capable of presenting 20 Hz and 30 Hz stimuli at a 60 Hz refresh rate. In contrast, the approximation approach theoretically can realize visual flickers at any frequency lower than half of the refresh rate. Therefore, the approximation approach can significantly facilitate the design and implementation of a high-frequency SSVEP-based BCI. An LED-based stimulator could be used to facilitate the direct comparison of the two approaches with high frequencies. This study shows that, within the alpha frequency band, there is no significant difference of the SSVEPs elicited by the approximation and consistent-period approaches. However, a high refresh rate (e.g., 120 Hz) could be more preferable for a high-frequency SSVEP-based BCI. Since the approximation approach uses two neighboring frequencies derived from the constant-period approach to approximate a flickering frequency, a high refresh rate can improve the stability of the high-frequency flickering stimuli by reducing the interval between the two neighboring frequencies. For example, in terms of amplitude and phase, a 22 Hz stimulus under the 120 Hz refresh rate (approximated by mixing 20 Hz and 24 Hz periods) is more stable than that under the 60 Hz refresh rate (approximated by mixing 20 Hz and 30 Hz periods).

### 4 Phase Coding

In addition to the approximation approach for frequency coding, phase coding is another efficient way to increase the number of visual stimuli in the SSVEP-based BCI [2], [39], [40]. Since the SSVEP is time-locked and phase-locked to the flickering stimulus, visual targets tagged by flickering signals at the same frequency but with different phases can be identified by detecting the phase of SSVEPs synchronized to the stimulus signals. Lee et al. (2010) implemented a phase-coded BCI system using SSVEPs with eight different phases at 31.25 Hz [41]. Furthermore, the frequency and phase mixed coding approach has been proposed and implemented in a recent study [25]. In addition to the amplitude and SNR, this study also measured the phase and latency of the SSVEPs elicited by the approximation approach and the constant-period approach under the 75 Hz and 120 Hz refresh rates. The results show that the phase and latency using the approximation approach are as stable as the constant-period approach. Therefore, the approximation approach could be extended to generate a stimulus sequence with a specified phase. In practice, Equation (1) can be revised by adding the initial phase *ϕ* to generate the stimulus sequence with a specified phase:
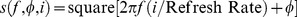
(9)


### 5 Potential Applications

The approximation approach for rendering SSVEP stimulus can be used to implement a practical BCI system that requires a large number of target selections and has potential to achieve a high ITR. With its capacity to present a large number of stimulus frequencies, the approximation approach can enable and facilitate various practical BCI applications such as an 8-target cursor system [36], a 12-target phone dialing system [13], and a 30-target spelling system [42]. In this way, the SSVEP-based BCI could lead to high BCI performance comparable to the BCI based on code modulation VEP (c-VEP) that requires a training procedure [14]. In addition, this method could be used to improve other compound stimulus design methods such as the dual-frequency stimulation method [43] and the frequency/phase mixed coding method [25]. Furthermore, the approximation approach provides a general framework to present SSVEP stimuli on the screen of mobile devices such as mobile phones and tablet computers [17]. A mobile visual stimulator can significantly improve the feasibility and practicality of the emerging mobile BCI technology [16]. In addition to various applications in BCIs, the approximation approach can also be used to facilitate the design of experiments that use the frequency-tagging technique with SSVEPs in the research of vision neuroscience. For example, the multiple flickering frequencies in feature selective attention [7] can be optimized to have maximum SNRs so that the attention-modulated SSVEP components can be more easily extracted for quantitative analysis.
